# Developmental mechanisms underlying webbed foot morphological diversity in waterbirds

**DOI:** 10.1038/s41598-020-64786-8

**Published:** 2020-05-15

**Authors:** Masayoshi Tokita, Hiroya Matsushita, Yuya Asakura

**Affiliations:** 1 Department of Biology, Faculty of Science, Toho University, 2-2-1 Miyama, Funabashi Chiba, 274-8510 Japan; 2 Present Address: Department of Polar Science, SOKENDAI (The Graduate University for Advanced Studies), 10–3 Midori-machi, Tachikawa Tokyo, 190–8518 Japan; 3 Present Address: Graduate School of Bioscience and Biotechnology, Fukui Prefectural University, 4–1–1 Kenjojima, Matsuoka, Eiheiji-cho Fukui, 910–1195 Japan

**Keywords:** Evolutionary developmental biology, Zoology

## Abstract

The webbed feet of waterbirds are morphologically diverse and classified into four types: the palmate foot, semipalmate foot, totipalmate foot, and lobate foot. To understand the developmental mechanisms underlying this morphological diversity, we conducted a series of comparative analyses. Ancestral state reconstruction based on phylogeny assumed that the lobate feet possessed by the common coot and little grebe arose independently, perhaps through distinct developmental mechanisms. *Gremlin1*, which encodes a bone morphogenetic protein (BMP) antagonist and inhibits interdigital cell death (ICD) in the foot plate of avian embryos, remained expressed in the interdigital tissues of webbed feet in the duck, common coot, little grebe, and great cormorant. Differences in *Gremlin1* expression pattern and proliferating cell distribution pattern in the toe tissues of the common coot and little grebe support the convergent evolution of lobate feet. In the totipalmate-footed great cormorant, *Gremlin1* was expressed in all interdigital tissues at St. 31, but its expression disappeared except along the toes by St. 33. The webbing of the cormorant’s totipalmate foot and duck’s palmate foot may have risen from distinct developmental mechanisms.

## Introduction

**Figure 1:**
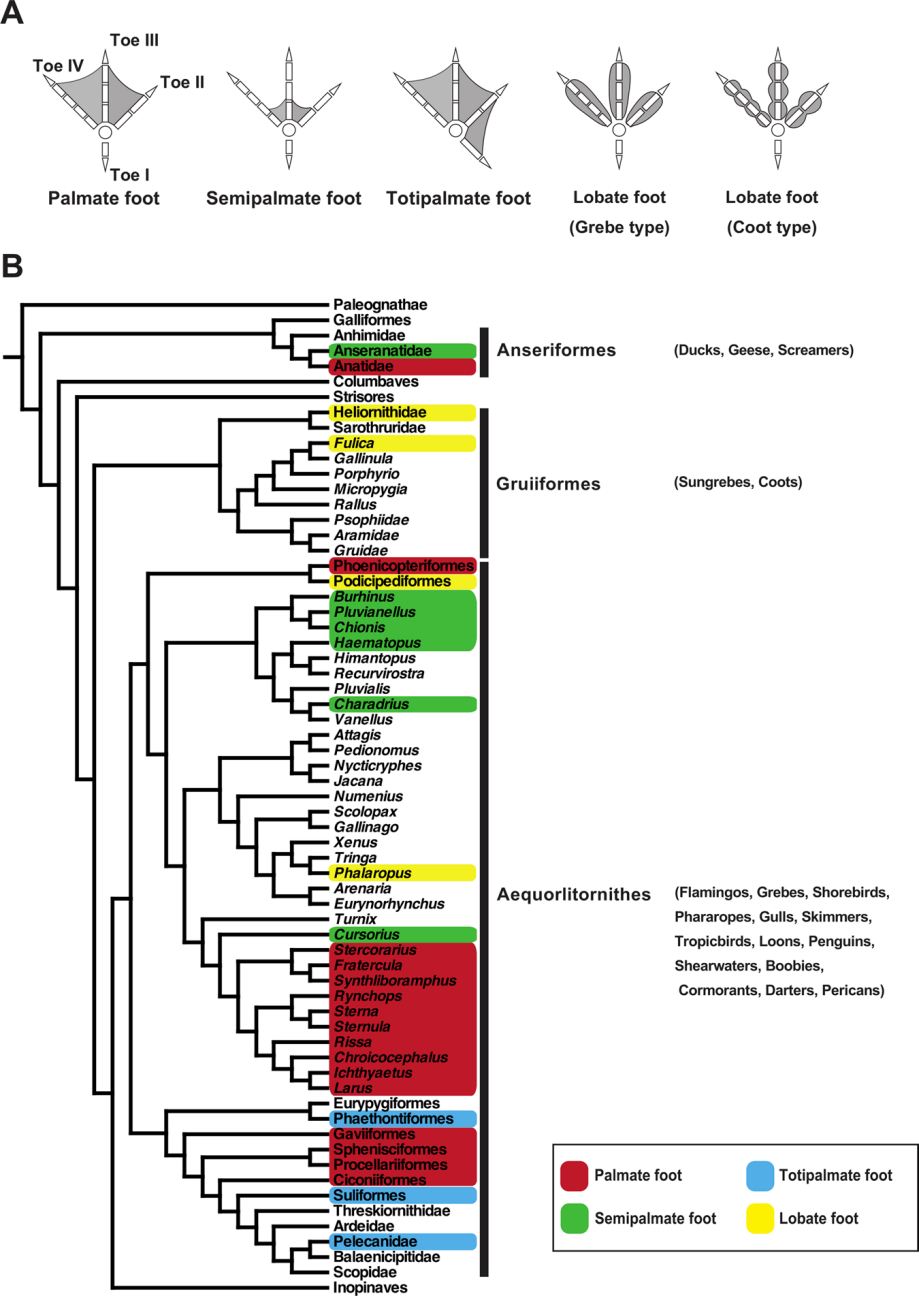
Morphological diversity of webbed feet in birds. (**A**) Simplified illustrations showing webbed foot types in birds. (**B**) Phylogenetic position of the waterbird taxa that possess webbed feet within the modern birds (Neornithes). Illustrations of avian feet in (**A**) were prepared by authors after modification of those in Evans (2016)^49^. Phylogenetic tree in (**B**) was prepared by authors using Mesquite 3.01^38^.

Waterbirds usually have webbed feet for foot-based propulsion. Webbed feet can be morphologically classified into four types: palmate, semipalmate, totipalmate, and lobate. Palmate feet are the most common type of webbed feet in waterbirds, where three front-facing toes (toes II, III, and IV) are entirely connected by webbing^1,2^ (Fig. 1A). Semipalmate feet have partial webbing at the base of the three front-facing toes (Fig. 1A). Totipalmate feet have a hallux connected by webbing to front-facing toe II, and toes II, III, and IV are entirely connected by webbing (Fig. 1A). The four toes of lobate feet are separated from each other, but each toe has leaf-like membranes (lobes) along the edges that produce propulsion in water^3^.

Webbed feet are observed in five clades of modern birds: (1) order Anseriformes including palmate-footed ducks and geese and semipalmate-footed magpie geese^1,4,5^; (2) order Gruiformes including lobate-footed sungrebes and coots^1,4,6^; (3) the clade composed of order Phoenicopteriformes including the palmate-footed flamingos and order Podicipediformes including the lobate-footed grebes^1,4,7^; (4) order Charadriiformes including the palmate-footed gulls, semipalmate-footed shorebirds, and lobate-footed genus *Phalaropes* in the family Scolopacidae^1,4,8^; (5) the large clade including the orders Eurypygiformes, Phaethontiformes, Gaviiformes, Sphenisciformes, Procellariiformes, Ciconiiformes, Suliformes, and Pelecaniformes with palmate (loons, penguins, and shearwaters) or totipalmate feet (Phaethontiformes and Suliformes)^1,4,9^. Webbed feet were acquired independently at least fourteen times in modern birds (Fig. 1B). To fully understand the origins and evolutionary history of webbed feet in birds, it is important to clarify the developmental mechanisms underlying webbed foot formation^10–13^.

Developmentally, the avian foot is derived from the digital plate of the hindlimb bud (i.e., foot plate) in embryos. The foot plate is composed of digital rays that give rise to future toes and the interdigital tissues that connects each of the digital rays^12–17^. During embryonic stages (St.) 30–35 based on the staging system for chickens^18^, interdigital tissue cells undergo apoptosis in a morphogenetic process called interdigital cell death (ICD)^15,19,20^. ICD contributes to the formation of free digits in the majority of terrestrial birds (e.g., chickens, pigeons, parrots, songbirds, etc.). The timing of ICD in embryogenesis is highly conserved among terrestrial bird species^12,18,21,22^. At St. 32–34 of embryogenesis, interdigital tissues become thinner and tissue regression begins. At St. 35, interdigital tissues are completely regressed. After the disappearance of the interdigital tissues, each toe is separated, and claws and toe pads form^18^.

Bone morphogenetic proteins (BMPs), a family of growth factors, facilitate ICD in vertebrates^23,24^. However, peridigital tissues around digital rays avoid ICD and give rise to tendons and other types of connective tissue^25^. In this area of the foot plate, two BMP antagonists, Gremlin and Noggin, inhibit BMP signalling and prevent ICD^10^. In duck embryos, the expression domain of Gremlin expands into all interdigital tissues^10^. Fibroblast growth factors (FGFs) are also known to inhibit ICD^24,26,27^. For example, FGF8 is expressed in the forelimb interdigital tissues of bat and dolphin embryos and inhibits ICD^28,29^.

Studies of foot development in ducks have partially uncovered the developmental mechanisms underlying palmate foot evolution^6,10,15,16,24,30^. However, the developmental mechanisms underlying the morphological diversity of webbed feet in waterbirds are far from being completely understood. Particularly few studies have reported on the molecular and cellular mechanisms that regulate lobate foot formation.

We carried out a series of comparative analyses to understand the diversification of webbed feet morphology in waterbirds. First, to understand the process of webbed foot evolution in birds, we conducted ancestral character state reconstruction based on the latest genome-based phylogeny of birds. Second, foot formation patterns were compared among several representative avian species (with and without webbed feet) to describe generality as well as diversity in avian webbed foot formation. Third, to understand the cellular mechanisms underlying morphological diversification of webbed feet, we described the expression pattern of *Gremlin1* and localisation of apoptotic cells within the interdigital tissues. Finally, to understand the cellular mechanisms underlying lobate foot evolution, proliferating cell distributions within toe tissues were compared among three bird species: the lobate-footed common coot and little grebe, and the non-lobate-footed common moorhen.

## Results

### Evolutionary history of webbed feet

**Figure 2:**
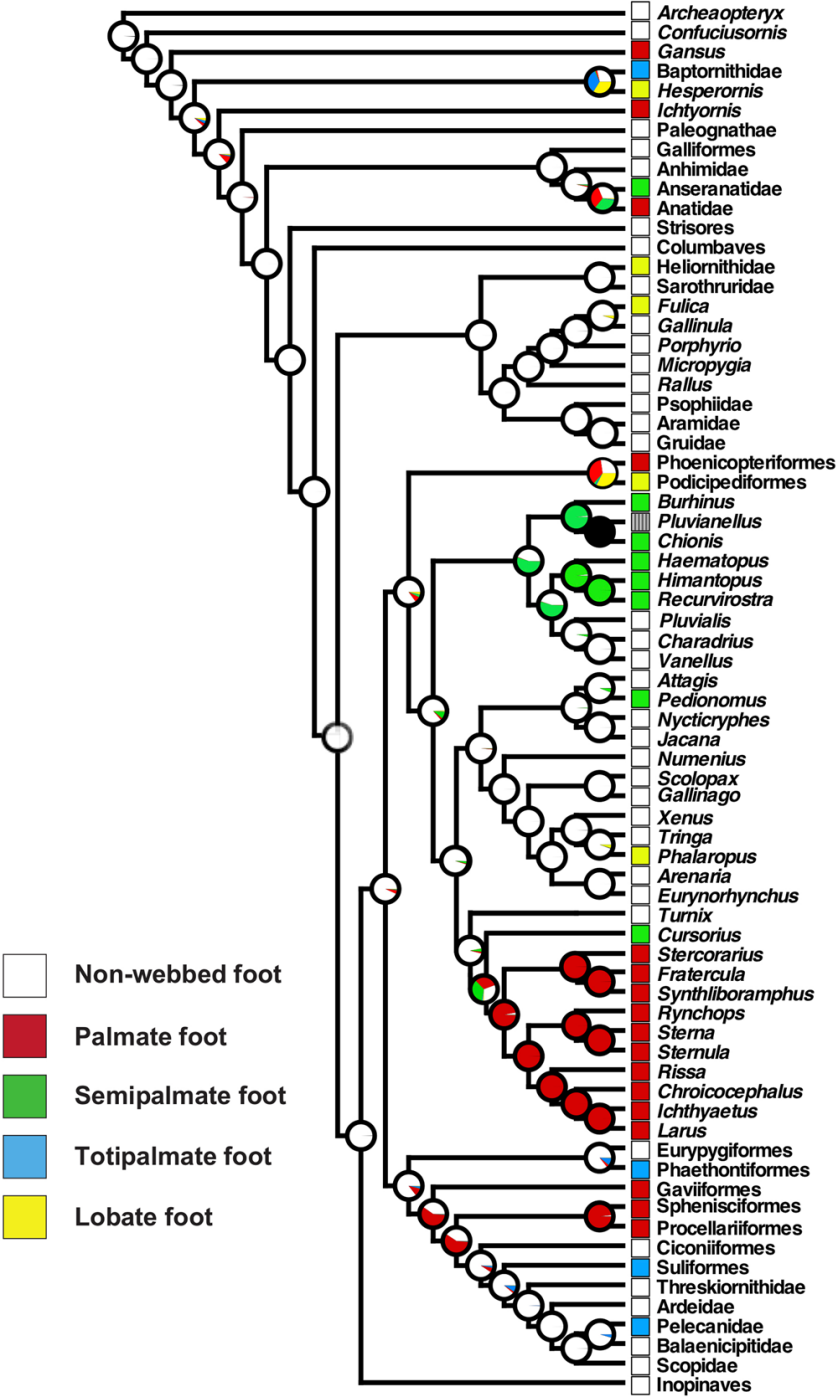
Reconstruction of webbed feet evolution in birds. Probable foot type possessed by the common ancestor is indicated by the pie charts at the nodes. Information about the foot morphology of *Pluvianellus* (Magellanic plover) belonging to the order Charadriiformes was unavailable in the references and its condition was indicated by a grey square with lines. Phylogenetic tree was prepared by authors using Mesquite 3.01^38^.

Through ancestral character state reconstruction of the phylogeny of birds, we suggest that webbed feet evolved as follows (Fig. 2). The common ancestor of Anseriformes possessed non-webbed feet. The family Anatidae (ducks) acquired their palmate feet after they diverged from Anseranatidae (magpie-geese). In Gruiformes, whose common ancestor possessed non-webbed feet (anisodactyl feet), the family Heliornithidae (finfoots) and the genus *Fulica* (coots) of the family Rallidae acquired their lobate feet in parallel probably as a secondary adaptation to aquatic environments. The common ancestor of the palmate-footed Phoenicopteriformes (flamingos) and lobate-footed Podicipediformes (grebes) likely possessed palmate feet (palmate 36 %, lobate 33%, non-webbed 27%, semipalmate 2%, totipalmate 2%). The common ancestor of Charadriiformes possessed anisodactyl feet but a complex nature of webbed feet evolution was apparent in this family. Palmate feet evolved once at the base of the suborder Lari. Semipalmate feet evolved at least three times: 1) at the common ancestor of the suborder Charadrii, 2) at the genus *Pedionomus* (plains-wanderer) of the suborder Scolopaci, 3) at the genus *Cursorius* (courser) of the suborder Lari. Lobate feet evolved once in the genus *Phalaropus* (phalarope) through modification of the ancestral anisodactyl feet. The common ancestor of the Gaviiformes, Sphenisciformes, Procellariiformes, Ciconiiformes, Suliformes, and Pelecaniformes possessed palmate feet. The common ancestor of the Ciconiiformes, Suliformes, and Pelecaniformes lost palmate feet and reacquired non-webbed feet. The totipalmate feet of Suliformes (boobies, frigatebirds, and cormorants) and Pelecanids (pelicans) might have evolved in parallel.

### Comparison of toe formation pattern and interdigital tissue regression

How and when ICD appears in foot morphogenesis was examined in embryos of the Japanese quail, mallard duck, little grebe, great cormorant, common moorhen, and common coot.

#### **Japanese quail (*Coturnix japonica*)** Anisodactyl foot

**Figure 3:**
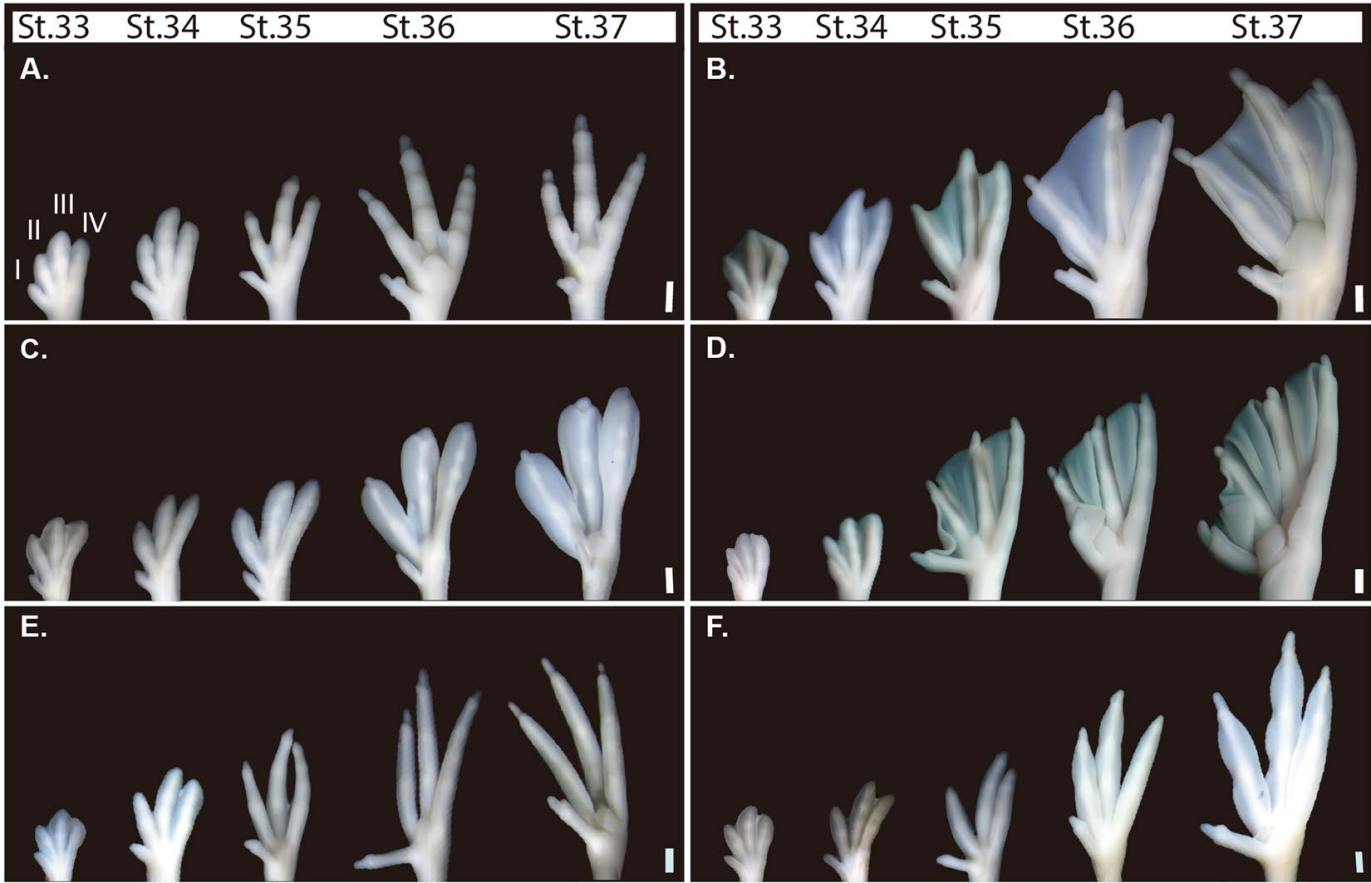
Interspecific comparison of foot morphogenesis. Left foot (ventral view) development was compared among the embryos of six species of birds at St. 33, 34, 35, 36, and 37. (**A**) Japanese quail. (**B**) Mallard duck. (**C**) Little grebe. (**D**) Great cormorant. (**E**) Common moorhen. (**F**) Common coot. Scale bars are 1 mm.

Interdigital tissues started to regress and each toe began to project distally during St. 33-34 (Fig. 3A). Interdigital tissues were completely lost at St. 35. Claws and toe pads were formed at St. 36.

#### **Mallard duck (*Anas platyrhynchos*)** Palmate foot

The distal edges of the interdigital tissues were slightly regressed during St. 33-34 (Fig. 3B). At St. 35, interdigital tissue remained between toes II and III, and toes III and IV, while toe I was clearly separated from toe II. After St. 36, when claws had already formed, interdigital tissues remained as webbing (Fig. 3B).

#### **Little grebe (*Tachybaptus ruficollis*)** Lobate foot

During St. 33-34, the distal edges of the interdigital tissues had a wedge-like slit (Fig. 3C). At St. 35, the broad area of interdigital tissues along toes II, III, and IV remained. After St. 36, interdigital tissues further expanded distally and toes II, III, and IV were accompanied with oar-like lobes.

#### **Great cormorant (*Phalacrocorax carbo*)** Totipalmate foot

At St. 33-34, the distal edges of the interdigital tissues were slightly depressed (Fig. 3D). At St. 35, most of the interdigital tissue remained between toes I and II, toes II and III, and toes III and IV. After St. 36, when the claws had already formed on all toes, interdigital tissues remained.

#### **Common moorhen (*Gallinula chloropus***) Anisodactyl foot

Like in terrestrial quail embryos, interdigital tissues were already regressed at St. 33,34 (Fig. 3E). Interdigital tissues were completely lost at St. 35. After complete separation of each toe, toe length was relatively longer than in the quail (Fig. 3A,E).

#### **Common coot (*Fulica atra*)** Lobate foot

During St. 33-34, interdigital tissues started to be regressed and outgrowth of each toe was observed (Fig. 3F). At St. 35, the interdigital tissues apparently disappeared. However, the relative width of toes II, III, and IV is slightly wider than in the moorhen (Fig. 3E,F). After St. 36, the width of toes II, III, and IV further increased, forming lobes along the toes. At St. 37, constrictions of the lobe tissues were observed at the phalanx joints.

### **Spatial pattern of*Gremlin1*expression and apoptosis in interdigital tissue**

**Figure 4:**
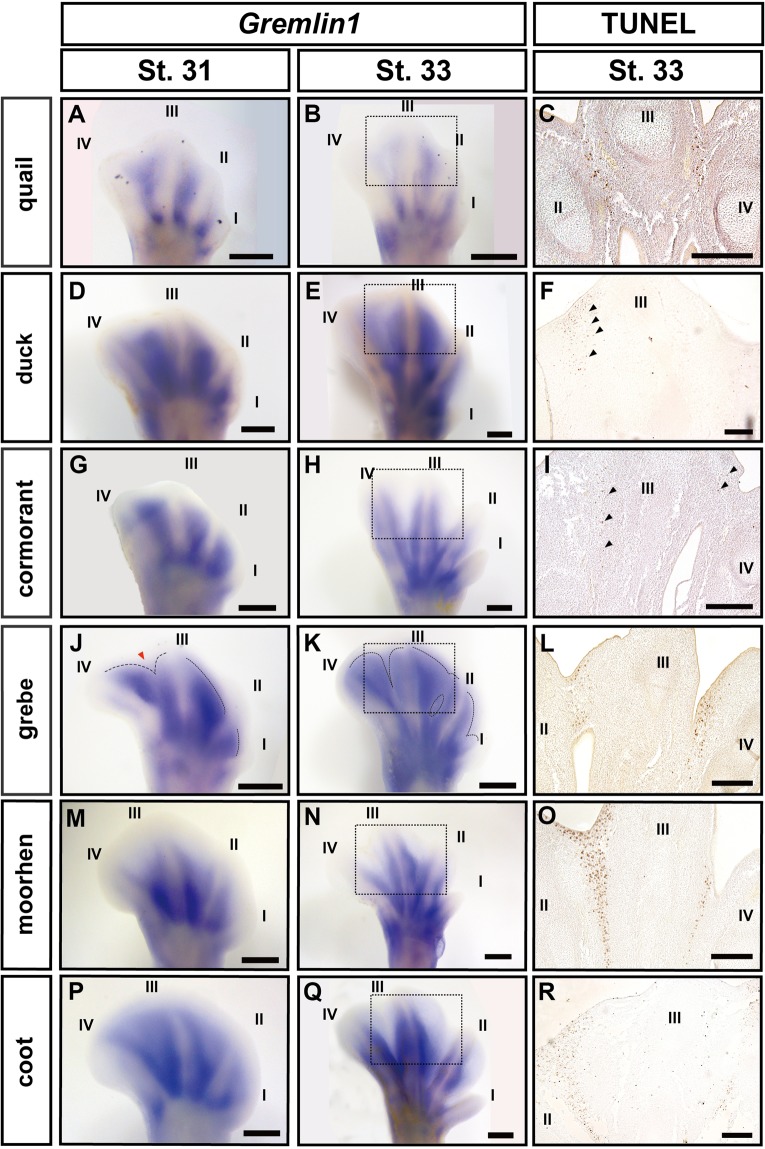
Interspecific comparison of spatial pattern of *Gremlin1* expression and apoptotic cells within interdigital tissues. Expression of *Gremlin1* in the embryonic foot at St. 31 and 33 of the Japanese quail (**A,B**), mallard duck (**D,E**), great cormorant (**G,H**), little grebe (**J,K**), common moorhen (**M,N**), and common coot (P, Q). Apoptotic cells were detected by TUNEL assay for Japanese quail (**C**), mallard duck (**F**), great cormorant (**I**), little grebe (**L**), common moorhen (**O**), and common coot (**R**) embryos at St. 33. Scale bars in A, B, D, E, G, H, J, K, M, N, P, Q are 0.5 mm. Scale bars in C, F, I, L, O, R are 0.2 mm.

In the quail embryos, *Gremlin1* was expressed in most of the proximal domain of the interdigital tissues at St. 31 (Fig. 4A), but its expression domain became relatively smaller at St. 33 (Fig. 4B) similar to results reported for chicken embryos^10^. In the duck embryos at St. 31-33, *Gremlin1* was expressed in most areas except for the distal edge of the interdigital tissues as reported by Merino *et al*. (1999)^10^ (Fig. 4D,E). In the cormorant embryos, *Gremlin1* was expressed in all interdigital tissues at St. 31 (Fig. 4G), but expression disappeared except for in the region adjacent to the toes at St. 33 (Fig. 4H). In St. 31 grebe embryos, *Gremlin1* was expressed in all interdigital tissues, but wedge-like *Gremlin1*-negative domains were detected in the disto-medial region of the interdigital tissues between toes III and IV (Fig. 4J). At St. 33, the *Gremlin1*-negative domain between toes III and IV expanded proximally and became slit-like in shape (Fig. 4K). Concurrently, the wedge-like *Gremlin1*-negative domains appeared between toes I and II, and II and III (Fig. 4K). In St. 31 moorhen embryos, V-shaped expression of *Gremlin1* was observed in the proximal region of the interdigital tissues between toes I and II, II and III, and III and IV (Fig. 4M). At St. 33, the edge of *Gremlin1* expression domains became clearer and localised along the toes (Fig. 4N). In the St. 31 embryos of the coot, which is lobate-footed and closely related to the moorhen (Fig. 2), fan-shaped expression of *Gremlin1* was observed in the broad region of interdigital tissues between toes I and II, II and III, and III and IV (Fig. 4P). At St. 33, *Gremlin1* was strongly expressed along the toes, as in the moorhen (Fig. 4Q). However, the width of *Gremlin1* expression domains along the toes in the coot embryos were wider than in similar stage moorhen embryos (Fig. 4N,Q).

The pattern of apoptotic cell distribution within the interdigital tissues of the embryonic foot was very different among the bird species examined. In the non-web-footed quail and moorhen, as well as in the lobate-footed grebe and coot, apoptotic cells were localised in the area of the interdigital tissues where *Gremlin1* was not expressed (Fig. 4C,L,O,R). In the palmate-footed duck and totipalmate-footed cormorant, a small number of apoptotic cells were detected within the interdigital tissues (Fig. 4F,I).

### Cell proliferation pattern in lobate foot formation

**Figure 5:**
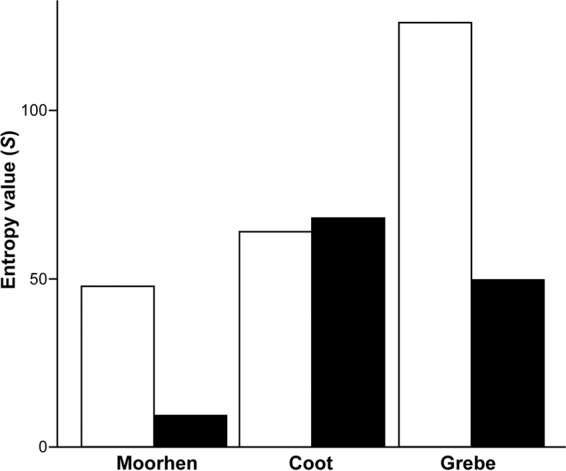
Degree of disorderliness of proliferating cell distributions in toes. Bar chart showing entropy values calculated from proliferating cell distributions in the transverse sections of the second joint (white bars) and the centre of the second phalanx (black bars) of toe III of St. 37 embryos of the common moorhen, common coot, and little grebe. Values represent the average of three individuals of each species. The entropy value indicates the degree of disorderliness of the proliferating cell distribution.

To understand the cellular mechanisms underlying lobe formation in the lobate feet, we compared the distribution pattern of phospho-histone H3-positive proliferating cells within toe tissues among three bird species: the lobate-footed common coot and little grebe, and the non-lobate-footed common moorhen. In the moorhen, the entropy value, which indicates the degree of disorderliness of the proliferating cell distribution in the tissues, estimated for the toe joint was about five times higher than that for the centre of the phalanx (Fig. 5). In the coot, which is closely related to the moorhen and possesses lobate feet, the pattern was different; the entropy value estimated for the centre of the phalanx was very slightly higher than that for the joint (Fig. 5). In contrast, in the grebe, which acquired lobate feet independently from the coot, the entropy value estimated for the toe joint was about double that for the centre of the phalanx (Fig. 5).

## Discussion

### Evolution and development of the lobate foot

Microscopic and histochemical analyses by Hurlé and Climent (1987)^6^ revealed that the lobes of coot feet secondarily outgrow from the toe margins after each toe is separated by ICD. Our inter-specific comparison of embryogenesis confirmed their result (Fig. 3F). The process of webbing formation in the coot is distinct from that in the palmate feet of ducks, where a broad region of the interdigital tissues lacking ICD gives rise to the final webbing^10^ (Fig. 4D,E,F). In the present study, we found that the width of *Gremlin1* expression domains along the toes in the embryos of the lobate-footed coot was wider than that of similar stage moorhen embryos without lobate feet (Fig. 4N,Q). We speculate that the lobes of coot feet are formed through two phases. First, the *Gremlin1* expression domains along the margins of toes II, III, and IV form the primordia of the lobes, and second, after ICD is complete, the lobe primordia outgrow through an increase or maintenance of cell proliferation rate (see below).

*Gremlin1* is expressed in the interdigital tissues of the embryonic grebe foot similarly to in coot embryos (Fig. 4J,K), despite these two species acquiring lobate feet independently. However, the expression domain is broader in the grebe. Formation of broader lobes (without constriction at the joints of the toes) in grebe lobate feet may relate to this wider expression domain of *Gremlin1*. We speculate that those *Gremlin1*-positive interdigital tissues in grebe embryo give rise to future lobes through isometric growth of the whole foot, not through allometric growth facilitated by secondary outgrowth of the lobe primordia (as implied in the common coot).

**Figure 6:**
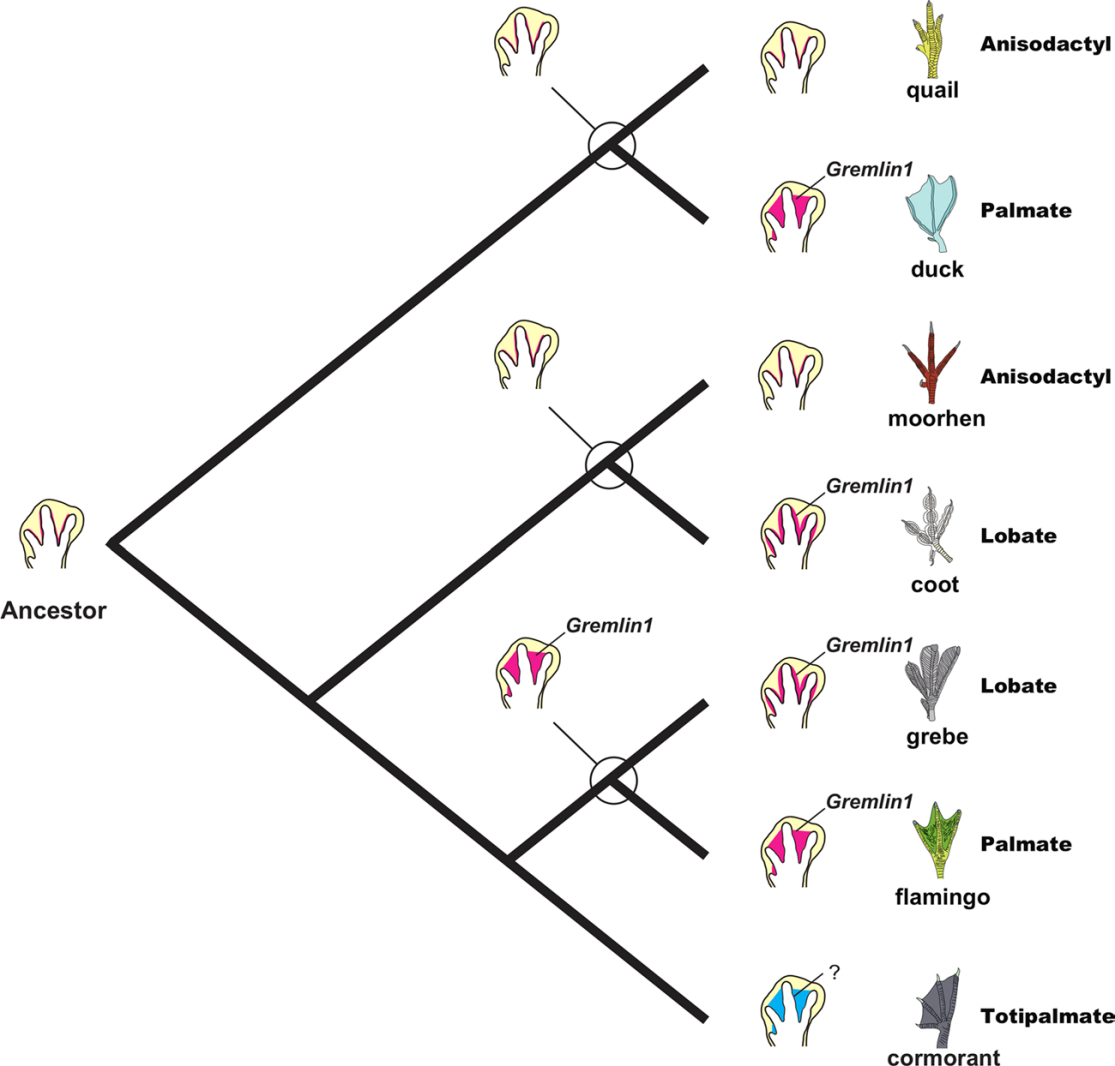
Scenario of webbed foot evolution in birds. The lobate feet observed in the common coot and little grebe were probably created through distinct developmental processes. The lobate foot of the common coot may have evolved through proliferation of interdigital tissue cells that express *Gremlin1* along the toes in an anisodactyl-footed ancestor. On the other hand, the little grebe’s locate foot may have arisen by loss of *Gremlin1* expression at the centre of the interdigital tissues of a palmate-footed ancestor. Although *Gremlin1* is expressed in all St. 31 interdigital tissues in the totipalmate-footed great cormorant, its expression disappears in the centre of the interdigital tissues at St. 33. This suggests that the webbing of the great cormorant may have arisen through a distinct developmental mechanism, where BMP signaling plays a fundamental role.

Ancestral state reconstruction suggested that the common ancestor of the common coot possessed anisodactyl feet, while the common ancestor of the little grebe possessed palmate feet (Fig. 2). These results suggest that the two types of lobate feet arose through distinct developmental processes (Fig. 6). The lobate foot of the common coot probably evolved through a proliferation of interdigital tissue cells that express *Gremlin1* along the toes of the anisodactyl ancestor. On the other hand, the lobate foot of the little grebe may have evolved through a contraction of *Gremlin1* expression at the centre of the interdigital tissues of the palmate-footed ancestor, where the gene was previously ubiquitously expressed.

The results of our analysis of proliferating cell distribution pattern in the toe tissues also supports the hypothesis above that the two types of lobate foot were acquired through distinct developmental processes. The proliferating cells were more randomly distributed in the toe joint than in the centre of the phalanx in moorhen and grebe embryos (Fig. 5). In contrast, the degree of disorderliness of the proliferating cell distributions were almost equivalent between the two positions in the coot (Fig. 5).

In bird embryos, the architectural structure of the toe skeleton and its accompanying tendons is almost complete by St. 35, but phalanges continue to grow proximodistally even after that stage^25^. Similarly, the dense connective tissues including epiphysial cartilage and tendon, joint cavity, and synovial membrane also continue to grow through cell proliferation after St. 35, ^25,31,32^.

Here, we considered the pattern of proliferating cell distribution observed in the moorhen to reflect the condition of all other bird species without webbed feet. A larger amount of dense connective tissue with a higher cell proliferation rate is localised at the toe joint, compared to the lower cell proliferation rate at the centre of toe phalanges. This could be a cause of the more random distribution of proliferating cells in the joints of the anisodactyl moorhen. The same pattern was observed in grebe embryos. The lobate foot of the grebe has oar-like lobes along front-facing toes (II, III, and IV). The much higher entropy values of both the joint and centre of the phalanx in the grebe may relate to the fact that the volume of dense connective tissue (with higher cell proliferation rate) forming the lobes is higher along the proximodistal axis of the toes in this species.

Unlike in the grebe’s lobate foot, the lobes in the coot foot are constricted at the toe joints forming a “pea-pod” shape (Fig. 1A). The pattern of proliferating cell distribution observed in coot toes may relate to this unique toe architecture. It is assumed that the amount of dense connective tissue with higher cell proliferation rate is higher in the centre of toe phalanges due to association of the lobes. This may reflect the result that the proliferating cells were slightly more randomly distributed in the centre of the phalanx than at the joint in coot toes. In fact, entropy values for the toe joints are not very different between the coot and moorhen. In conclusion, the two types of lobate foot possessed by the grebe and coot are probably acquired through different developmental processes including regulatory changes in *Gremlin1* expression and cell proliferation rate in palmate-footed and anisodactyl ancestors, respectively. At the same time, we need to be careful in leading to a conclusion about the process of lobate feet evolution because phylogenetic position of grebes could be changed depending on molecular phylogeny. If we use genome sequence-based phylogenies by Jarvis *et al*.^33^ and Kimball *et al*.^34^ where grebes are more closely related to Columbiformes (pigeons and sandgrouse) (not to Charadriiformes as in Prum *et al*.^4^), the foot morphology of the ancestor of grebes becomes unclear: anisodactyl or palmate or lobate feet (Supplementary Figure S1).

### Evolution and development of the totipalmate foot

In St. 31 embryos of the totipalmate-footed cormorant, *Gremlin1* was expressed in all hindlimb interdigital tissues (Fig. 4G). However, its expression disappeared in the centre of the interdigital tissues of St. 33 embryos (Fig. 4H). This suggests that the webbing in the totipalmate foot is brought about by a developmental mechanism distinct from that of the duck’s palmate foot (Fig. 4D,E; Fig. 6). Merino *et al*.^24^ showed that implantation of fibroblast growth factors (FGFs)-soaked beads into interdigital tissue inhibits ICD in birds. Also, some authors reported that *Fgf8* is expressed in the interdigital tissues of the embryos of bats and cetaceans that formed wings and flippers, respectively^28,29^. Regulatory change of FGF signalling could be a potential mechanism underlying avoidance of the interdigital tissue cell death in the totipalmate-footed cormorants.

Ancestral state reconstruction suggested that the common ancestor of the Gaviiformes, Ciconiiformes, Sphenisciformes, Procellariiformes, Suliformes, and Pelecaniformes possessed palmate feet, but that the common ancestor of the clade composed of Ciconiiformes, Suliformes, and Pelecaniformes secondarily reacquired non-webbed feet (Fig. 2). The analysis also indicated that the totipalmate feet of Suliformes (including the great cormorant and their relatives), Pelecaniformes (pelicans and their relatives), and Phaethontiformes (tropicbirds and their relatives) were acquired independently and their totipalmate feet arose from “non-webbed” anisodactyl feet, not from the palmate feet. When complex morphological structures are made through highly complicated developmental process and then lost, reacquisition of the trait is generally rare^35^. However, there are some examples where the trait was reacquired through new developmental mechanisms^11,36^. If the palmate feet and the totipalmate feet are formed through distinct molecular mechanisms, successive evolution of the totipalmate feet from the palmate feet (and vice versa) might not be prerequisite. To identify whether totipalmate-footed birds share foot developmental mechanisms, examination of pelecaniform and phaethontiform embryogeneses would be also important.

## Methods

### Ancestral character state reconstruction

Following Ksepka *et al*.^37^, ancestral character state reconstruction of foot morphology was conducted using Mesquite 3.01^38^ under the MK1 Maximum Likelihood model applied to an unscaled phylogenetic tree. Foot types were scored as follows: “0” for non-webbed, “1” for palmate, “2” for semipalmated, “3” for totipalmate, and “4” for lobate feet. Data on the foot morphology of each taxon were derived from the references^1,2,39^.

We used a composite phylogeny of extant bird families prepared based on the phylogenies by the authors^4,8,40–44^ to map character states, because the recent genome sequence-based phylogeny of birds^4^ did not show the phylogenetic position of some web-footed species (e.g., the lobate-footed genera *Fulica* and *Phalaropus*). In this analysis, we treated *Phalaropus* as the sister group of the genus *Tringa*, considering phylogenetic tree of the order Charadriiformes by Fain and Houde (2007)^45^.

### Collection and staging of embryonic samples

Fertilised eggs of the little grebe (*Tachybaptus ruficollis*), common moorhen (*Gallinula chloropus*), common coot (*Fulica atra*), and great cormorant (*Phalacrocorax carbo*) were collected during 2016–2018, under permits issued from Gunma, Ibaraki, Chiba, Hyogo, and Yamanashi Prefectures. Fertilised Japanese quail (*Coturnix japonica*) and mallard duck (*Anas platyrhynchos*) eggs were purchased commercially. All eggs were incubated at 37 °C in the laboratory. Embryo staging was performed following Hamburger and Hamilton (1951)^18^. Animal experiments were conducted in accordance with the guidelines of Toho University (Regulations for Animal Experiments and Related Activities at Toho University), with approval of the Committee on the Ethics of Animal Experiments of Faculty of Science, Toho University (15–51–301, 16–52–301, 17–53–301, and 18–54–301).

### **Cloning and RNA probe synthesis of*Gremlin1***

Total RNA was extracted from the embryos using the NucleoSpin RNA kit (MACHEREY- NAGEL). RT–PCR was performed to amplify the fragments of *Gremlin1* mRNA in all bird species assessed. The primer sequences used for isolation of the fragments were Fw-5′-ATGGTCCGCACACTGTTGCC-3′ and Rv-5′-GCATTTGCCGTCACATGATGCTT-3′. The fragments were subcloned using the pGEM-T Easy Vector Systems (Promega) and DH5α competent cells (TOYOBO) and sequenced using Sanger sequencing. Sequence data were compared to a BLAST search to identify homologues of the isolated fragments. For synthesis of digoxigenin (DIG)-labelled RNA riboprobes, DNA templates with Sp6 and T7 promoters were first generated by PCR. Then, the templates were transcribed with an appropriate RNA polymerase.

### **Whole-mount*in situ*hybridisation**

Right hindlimb buds were excised from St. 31 and 33 embryos fixed with 4% paraformaldehyde in PBS and then preserved in 100% methanol at −30 °C. The tissues were rehydrated through a descending methanol series followed by two washes with PBT. The samples were permeabilised with 0.2 N hydrochloric acid in PBS for 10 min and with 20 μg/ml proteinase K in PBS for 30 min at 37 °C, before being washed with PBT for 10 min three times. Subsequently, the samples were fixed with 4% paraformaldehyde in PBS, washed with PBT three times, washed with prehybridisation buffer (50% formamide, 2x SSC, 1x Denthart’s solution, 100 μg/ml tRNA, 10 mM DTT, 0.1% Tween) once, and incubated in fresh prehybridisation buffer overnight at 65 °C. The prehybridisation buffer was then replaced with hybridization buffer (prehybridization buffer with 1 ug/ml of sodium dextran sulfate and DIG-labelled RNA riboprobe) and incubated over two nights at 65 °C.

Following hybridisation, the samples were washed with wash buffer (50% formamide, 2 x SSC, 0.1% Tween) for 30 min twice at 65 °C, again at 37 °C, and then for 30 min three times with 2x SSC at room temperature. To prevent non-specific binding of antibodies to the samples, samples were blocked with blocking buffer (Product no. 11096176001, Roche) for 1 hr at room temperature. The digoxigenin-labeled riboprobe signals were detected by incubation of the samples with Anti-Digoxigenin-AP Fab fragments (Product no. 11093274910, Roche) in blocking buffer at a concentration of 1/2000 at 4 °C overnight. After incubation, the samples were washed with distilled water first for 5 min three times at room temperature and then with PBT for 5 min three times at room temperature. The samples were preserved in PBT overnight at 4 °C and washed with TMN (100 mM NaCI, 100 mM Tris-HCI, 50 mM MgCl) for 5 min once at room temperature. Antibody detection was performed by incubating the samples with detection solution (10% polyvinyl alcohol (PVA)/TN with 50% TMN, 0.3% NBT and 0.4% BCIP) for 1–1.5 hr. The reaction was stopped with water. Samples were then washed once with PBT for 1 hr at room temperature, once with 100% ethanol for 20 min at room temperature, and for 5 min three times with PBT. Finally, the samples were post-fixed with 4% paraformaldehyde in PBS, and washed once with PBT.

### Detection of apoptotic cells

The embryos were fixed with 4% paraformaldehyde in PBS, washed with PBT three times at 4 °C, and dehydrated through an ascending methanol series in PBT (30%, 60%, 90%, 100%). The whole left hindlimb of each embryo was embedded in paraffin and sectioned into 8 μm thick slices using a microtome. To detect apoptotic cells in the foot tissues, the terminal deoxynucleotidyl transferase-mediated dUTP nick end labelling (TUNEL) assay was conducted using the *in situ* Apoptosis Detection Kit (TaKaRa) following the manufacturer’s protocol.

### Detection of proliferating cells

St. 37 embryos of the common coot, common moorhen, and little grebe were fixed with Serra’s fixative (ethanol: formalin: acetic acid = 6:3:1) overnight. The whole left hindlimb was excised and dehydrated using an ethanol series, placed in methyl benzoate and benzene, embedded in paraffin, and sliced transversely at a thickness of 10 μm. To count the number of proliferating cells within the hindlimb tissue, we performed immunohistochemistry using rabbit anti-phospho-histone H3 (PHH3) polyclonal antibody (1:40, Product no. PA5–40141, Invitrogen) as the primary antibody, and Alexa Fluor 488 goat anti-rabbit IgG (1:200, Invitrogen) as the secondary antibody. Sections were counterstained with a fluorescent blue nuclear stain (Hoechst; Product no. 343–07961, DOJINDO). Pictures of immunostained transverse sections were taken using a confocal microscope (Nikon, A1) (Supplementary Figure S2).

A method for quantitatively comparing the disorderliness of proliferating cell distributions in transverse sections has not been established yet. Therefore, we developed an equation that calculates an entropy value based on the sum of the logarithm of distances between proliferating cells. Distances between proliferating cells were caclulated based on the distance between black pixels in a binary image (e.g., monochrome image) following Sato and Suganuma (2013)^46^. The distribution of proliferating cells at the second joint and centre of the second phalanx of the third toe was quantified in the grebe, moorhen, and coot embryos. Three individuals were examined for each species. Three sections of the second joint and three sections of the centre of the second phalanx were examined for each individual. Entropy value should be high in the transverse sections where proliferating cells are distributed randomly and low in transverse sections where proliferating cells are distributed regularly.

To calculate the entropy value, we first prepared two pictures of each transverse section for one specimen of each species, one stained blue by Hoechst and another stained green by PHH3/Alexa 488. A binarised image of each picture was created following the Otsu method^47^. In binarised images, pixels with blue or green colour were treated as “1”, while pixels without colour were treated as “0”. The following equation was used to obtain the entropy value *S* from the binarised image in pixel size (m, n).

$$S=\mathop{\sum }\limits_{i=1}^{m}\mathop{\sum }\limits_{j=1}^{n}\mathop{\sum }\limits_{k=1}^{m}\mathop{\sum }\limits_{l=1}^{n}{Z}_{i,j}\cdot {Z}_{k,l}\cdot \,\mathrm{ln}\left(\frac{\sqrt{{(k-i)}^{2}+{(l-j)}^{2}}}{{W}_{max}}\right)$$

Zi,j and Zk,l represent the colour values (1 or 0) of pixels (i,j) and (k,l), respectively. The equation takes pixel pairs in which both Zi, j and Zk,l are 1, and calculates the distance between the pairs. The entropy value has conventionally been calculated by summing the distances between all pixel pairs. However, the shape of the toes, width of the joint section, and centre of the phalanx are different among the coot, moorhen, and grebe. Consequently, the entropy value calculated from wider sections tends to be larger than the value calculated from narrower sections. This means that the entropy value calculated from the second joint of the third toe and the value calculated from the centre of the second phalanx of the third toe cannot be compared directly in these bird species. Therefore, we also measured the maximum width (W_max_) of the second joint of the third toe. To normalise the entropy value of each section, the distance between pixel pairs was divided by the W_max_ of the section that contains the pixels. Calculations were performed using the programming language Python 3.6^48^.

## Supplementary information

Supplementary Figures.

Supplementary Figure Legends
