# Supplementary material for: Developmental mechanisms underlying webbed foot morphological diversity in waterbirds
Source: Sci Rep. 2020 May 15;10:8028. doi: 10.1038/s41598-020-64786-8 (PMC7229147; doi:10.1038/s41598-020-64786-8)
Supplement: Supplementary file 1 — Supplementary Figures. [file 41598_2020_64786_MOESM1_ESM.pdf]

Supplementary Information

Article title:

Developmental mechanisms underlying webbed foot morphological diversity in waterbirds

Authors:

Masayoshi Tokita, Hiroya Matsushita, and Yuya Asakura

(Toho University, Japan)

Supplementary Fig. S1

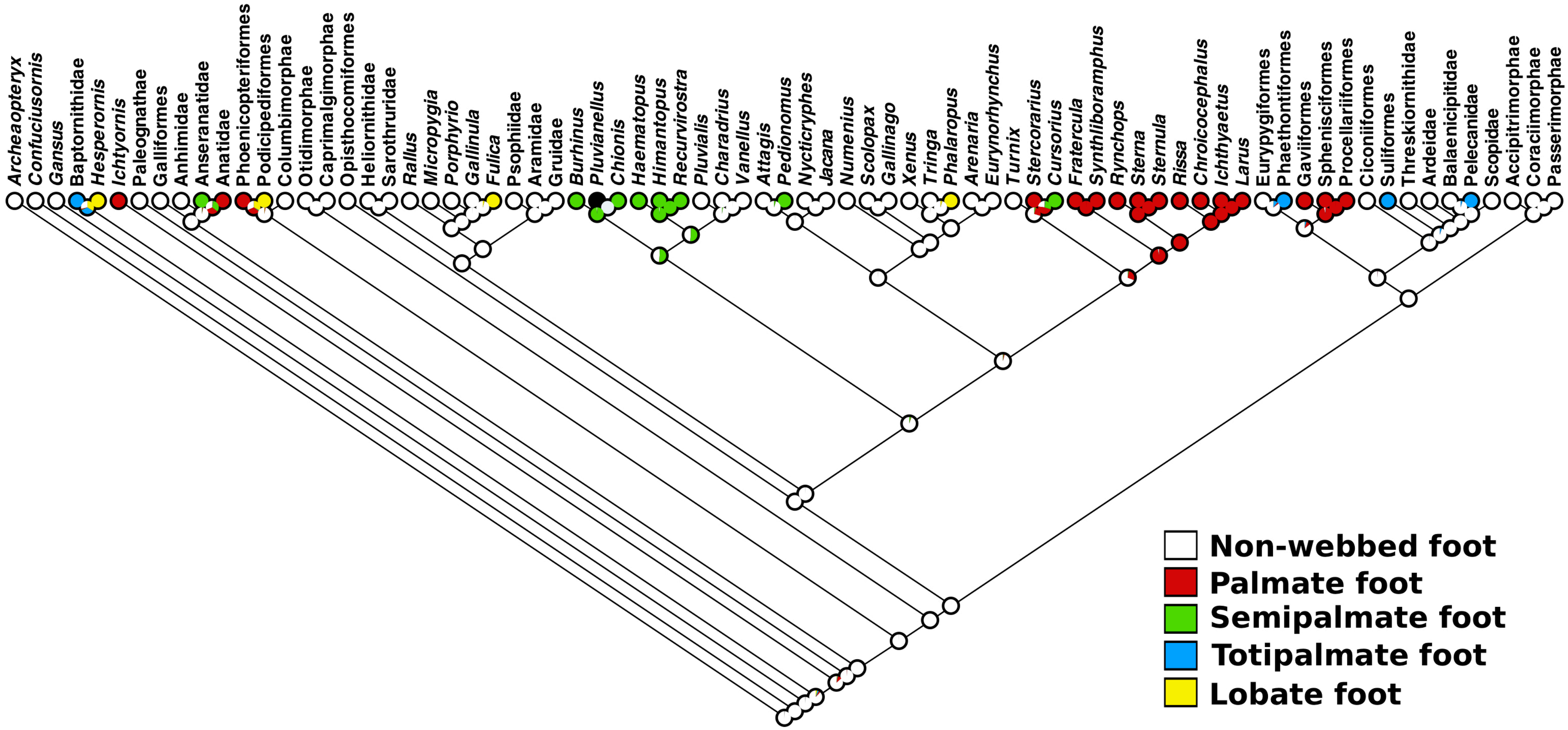

Developmental mechanisms underlying webbed foot morphological diversity in waterbirds

Masayoshi Tokita, Hiroya Matsushita, Yuya Asakura

Supplementary Fig. S2

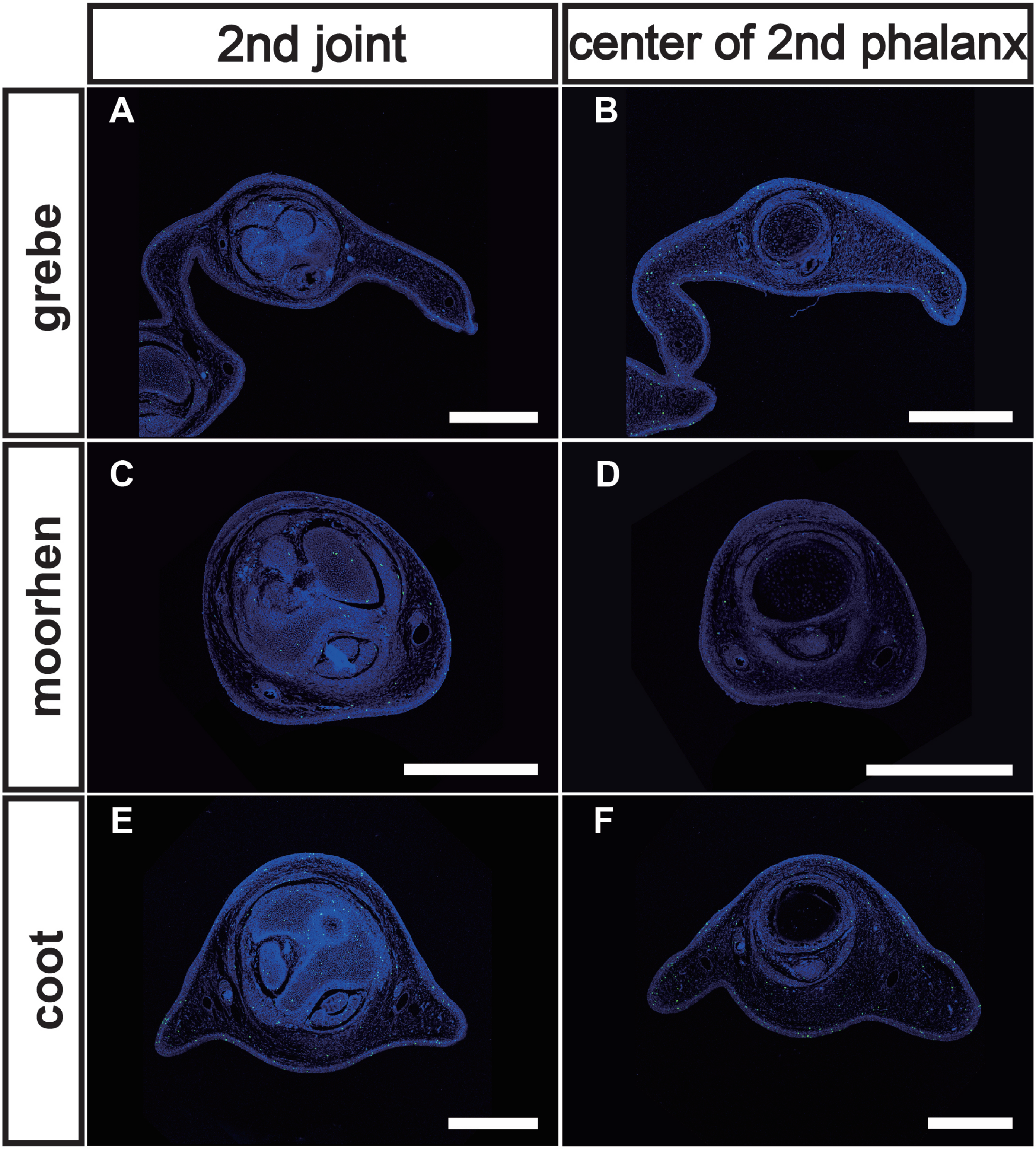

Developmental mechanisms underlying webbed foot morphological diversity in waterbirds
Masayoshi Tokita, Hiroya Matsushita, Yuya Asakura
