# Supplementary material for: Developmental mechanisms underlying webbed foot morphological diversity in waterbirds
Source: Sci Rep. 2020 May 15;10:8028. doi: 10.1038/s41598-020-64786-8 (PMC7229147; doi:10.1038/s41598-020-64786-8)
Supplement: Supplementary file 2 — Supplementary Figure Legends [file 41598_2020_64786_MOESM2_ESM.docx]

**Supplementary Figure S1. Reconstruction of webbed feet evolution in birds based on a composite phylogeny where genome sequence-based phylogeny by Jarvis *et al*. (2014) was used as a backbone.** Probable foot type possessed by the common ancestor is indicated by the pie charts at the nodes. Phylogenetic tree was prepared by authors using Mesquite 3.01 (Maddison and Maddison, 2003).

**Supplementary Figure S2. Interspecific comparison of proliferating cell distribution pattern**

Transverse sections of the second joint and centre of the second phalanx of toe III in St. 37 embryos of the little grebe (**A**, **B**), common moorhen (**C**, **D**), and common coot (**E**, **F**). Proliferating cells are labeled by anti-PHH3 antibody (green fluorescence). Scale bars are 0.5 mm.
